# Intravenous Remifentanil versus Epidural Ropivacaine with Sufentanil for Labour Analgesia: A Retrospective Study

**DOI:** 10.1371/journal.pone.0112283

**Published:** 2014-11-11

**Authors:** Rong Lin, Yiyi Tao, Yibing Yu, Zhendong Xu, Jing Su, Zhiqiang Liu

**Affiliations:** Department of Anaesthesiology, Shanghai First Maternity and Infant Hospital, Tongji University School of Medicine, Shanghai, China; The James Cook University Hospital, United Kingdom

## Abstract

Remifentanil with appropriate pharmacological properties seems to be an ideal alternative to epidural analgesia during labour. A retrospective cohort study was undertaken to assess the efficacy and safety of remifentanil intravenous patient-controlled analgesia (IVPCA) compared with epidural analgesia. Medical records of 370 primiparas who received remifentanil IVPCA or epidural analgesia were reviewed. Pain and sedation scores, overall satisfaction, the extent of pain control, maternal side effects and neonatal outcome as primary observational indicators were collected. There was a significant decline of pain scores in both groups. Pain reduction was greater in the epidural group throughout the whole study period (0∼180 min) (P<0.0001), and pain scores in the remifentanil group showed an increasing trend one hour later. The remifentanil group had a lower SpO_2_ (P<0.0001) and a higher sedation score (P<0.0001) within 30 min after treatment. The epidural group had a higher overall satisfaction score (3.8±0.4 vs. 3.7±0.6, P = 0.007) and pain relief score (2.9±0.3 vs. 2.8±0.4, P<0.0001) compared with the remifentanil group. There was no significant difference on side effects between the two groups, except that a higher rate of dizziness (1% vs. 21.8%, P<0.0001) was observed during remifentanil analgesia. And logistic regression analysis demonstrated that nausea, vomiting were associated with oxytocin usage and instrumental delivery, and dizziness was associated to the type and duration of analgesia. Neonatal outcomes such as Apgar scores and umbilical-cord blood gas analysis were within the normal range, but umbilical pH and base excess of neonatus in the remifentanil group were significantly lower. Remifentanil IVPCA provides poorer efficacy on labor analgesia than epidural analgesia, with more sedation on parturients and a trend of newborn acidosis. Despite these adverse effects, remifentanil IVPCA can still be an alternative option for labor analgesia under the condition of one-to-one bedside care, continuous monitoring, oxygen supply and preparation for neonatal resuscitation.

## Introduction

Epidural analgesia is efficient to relieve labour pain with fewer side effects on parturients and neonatus and regarded as the gold standard for obstetric analgesia [1]. However, some certain clinical conditions restrict its administration, such as maternal rejection or noncooperation, coagulation disorders, infection or tumor close to site of puncture, allergic reaction to local anesthetic, and spinal deformity [2]. It is clear that an effective and safe alternative should be established.

Remifentanil for intravenous patient-controlled analgesia (IVPCA) seems to be a promising option because of its particular pharmacokinetic and pharmacodynamic characteristics. Remifentanil as an ultra short-acting synthetic opioid has a very fast onset time (30∼60 s), peak analgesic effect of 2.5 min, a high metabolic rate (context-sensitive half-life about 3∼4 min), and no accumulated effect with repeated or long-term use [3]–[5]. Although it crosses the placental barrier with no difficulty, the drug can be degraded rapidly in the foetus [6].

A lot of studies with respect to the efficacy and complications of remifentanil for labour analgesia have been carried out. A prospective, randomised study from Douma et al. [7] on a group of only 20 patients discovered superior anesthetic effect was provided by epidural analgesia compared with remifentanil IVPCA. Volmanen et al. [8] designed a controlled, double-blinded study (42 parturients were randomly recruited) to observed analgesic efficacy of remifentanil and epidural analgesia just lasting for 60 min during the first stage of labour, and also reached similar conclusions. But they only evaluated fetal heart rate (FHR), umbilical artery pH and 1 min Apgar scores as fetal outcomes. Another randomised, controlled trial of Tveit et al. [9] (EA group 20, RA group 17) reported that remifentanil was more likely to cause sedation and oxygen desaturation, but was safe to neonates. In our recent meta-analysis involving 5 studies, remifentanil IVPCA was not found to afford better pain relief than epidural analgesia, but it did not bring serious adverse outcomes to mother and newborn [10]. Since most of these studies were somewhat limited by small sample sizes, a short observation period or inadequate assessment, it still remains controversial whether we can administrate remifentanil during labour without worry.

Thus we conducted this large sample study to retrospectively investigate maternal and neonatal outcomes of remifentanil IVPCA compared with epidural analgesia.

## Materials and Methods

**Figure 1:**
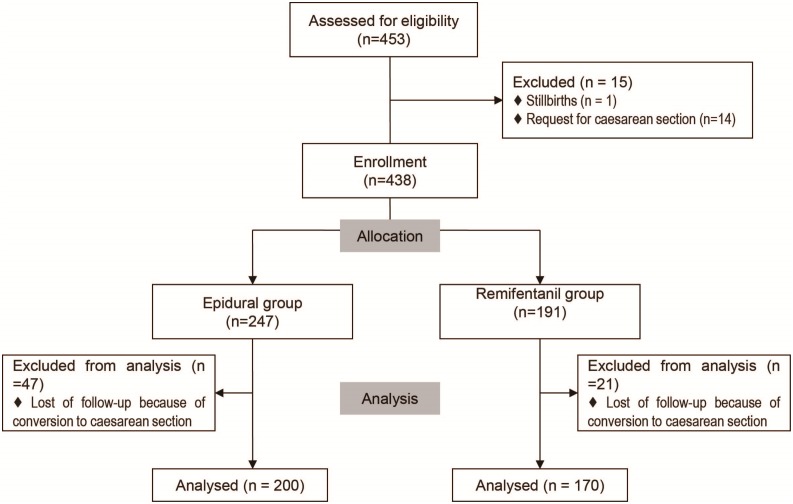
Flow-process diagram of the retrospective study.

The study obtained approval from the Research Ethics Committee of Shanghai First Maternity and Infant Hospital. Written consent was obtained from each patient. All electronic medical records of parturients who had accepted intravenous remifentanil or epidural analgesia during labour in our institution from January 2013 to July 2013 were reviewed. Inclusion criteria were as follows: primipara (ASA status I or II), singleton pregnancy with cephalic presentation, gestational age of >36 weeks, spontaneous or induced labour. Records of women with request for caesarean section or stillbirth were excluded. In light of analgesia technique the eligible parturients elected, they were divided into two groups: the remifentanil group and the epidural group (Fig. 1).

### Intravenous remifentanil analgesia regimen

Parturients were directed how to operate the PCA pump (Baxter 6060 Multi-Therapy infusion pump, Baxter Healthcare Corporation, Kista, Sweden) before the start of analgesia. The dosage regimen of remifentanil hydrochloride (Ultiva, GlaxoSmithKline, Oslo, Norway) diluted with saline to a concentration of 20 µg·ml^−1^ was set to PCA bolus of 0.4 µg·kg^−1^, a continuous background infusion at 0.04∼0.05 µg·kg^−1^·min^−1^ and a lockout time of 5 min. PCA doses were calculated according to estimated bodyweight (body height in centimeters -100) [9], [11], [12].

### Epidural analgesia regimen

An epidural catheter was inserted in the epidural space at L2–3 or L3–4 with the patient in a lateral decubitus position by an anaesthesiologist. Then the same PCA pump was connected to the cannula. Parturients received a 10 ml initial loading dose of 0.068% ropivacaine and 0.3 µg·ml^−1^ sufentanil, followed by a maintenance dose at 8 ml·h^−1^ and a PCA bolus dose of 5 ml with a 15-min lockout interval. If necessary, the infusion dose could be adjusted (5–10 ml·h^−1^).

One-to-one nursing service was provided to every parturient entering into the delivery room. The analgesia was applied when the cervix dilated to 3 cm, and terminated before the beginning of the second stage of labor. Before administration of analgesia, intravenous infusion of lactated Ringers solution and oxygen inhalation through nasal tube were given by convention. Routine monitoring, including maternal noninvasive blood pressure (NIBP), heart rate (HR), pulse oxygen saturation (SpO_2_), uterine activity and FHR by external tocodynamometry were accomplished and measured continuously. Numerical rating scale (NRS) pain score (an 11-point scale, 0 = no pain and 10 = worst pain imaginable) was used to assess the pain level. And the evaluation of sedation referred to the Ramsay sedation score (1 = anxious, agitated, restless; 2 = cooperative, oriented, tranquil; 3 = responds to simple commands only; 4 = brisk response to light glabellar tap or loud auditory stimulation; 5 = sluggish response to light glabellar tap or loud auditory stimulation; 6 = no response to light glabellar tap or loud auditory stimulus). Non-invasive measurements mentioned above as well as pain and sedation scores were recorded before and immediately after treatment, afterwards every 30 min.

The day after delivery, parturients were asked about their overall satisfaction with analgesic therapy on a five-point verbal rating scale (0 = very dissatisfied, 1 = dissatisfied, 2 = neutral [neither satisfied nor dissatisfied], 3 = satisfied, 4 = very satisfied), and to express the degree of pain relief likewise on a 5-point categorical scale (0 = very poor, 1 = poor, 2 = moderate, 3 = good, 4 = very good) [9].

Excepting above, other data collected included: maternal demographic characteristics (age, gestational weeks, height, weight, BMI), delivery mode, oxytocin treatment, durations of analgesia, maternal adverse reactions (respiratory depression, excessive sedation, nausea and vomiting, skin pruritus), neonatal outcomes (Apgar scores at 1 and 5 min, umbilical-cord blood gas analysis, requirement for resuscitation).

### Statistical analysis

SPSS version 18.0 (SPSS Inc., Chicago, IL, USA) was used for all data analysis. As a general rule, data were expressed as mean ± SD or frequency (percentage). P values less than 0.05 were considered statistically significant. Continuous variables were processed with Student’s *t*-test. And Chi-square test was performed for categorical variables. To evaluate the relationship of side effects to other factors, logistic regression analysis was performed.

## Results

Through reviewing the database, we identified 453 medical records that met inclusion criteria. Among them, fifteen subjects were ruled out on account of stillbirth (n = 1) or request for caesarean section (n = 14). Follow-ups of sixty-eight parturients were brought to a close due to their conversion to caesarean section. Finally, the following analysis was on the base of 370 observations (Epidural group 200, Remifentanil group 170) (Fig. 1). The conversive rate of caesarean section in the epidural group is significantly higher than in the remifentanil group (19.0% vs. 11.0%, P = 0.021). But there was no statistically significant difference between the two groups with regard to the indications for cesarean section (Table 1).

**Table 1:** Maternal demographic characteristics and labour data.

	Epidural Group (n = 200)	Remifentanil Group (n = 170)	P value
**Age (years)**	29.3±3.1	29.6±3.2	0.208
**Gestational age (weeks)**	39.6±1.1	39.6±1.0	0.817
**Height (cm)**	1.60±0.03	1.61±0.03	0.024
**Weight (kg)**	71.0±5.3	70.1±8.6	0.373
**BMI (kg·m^−2^)**	27.3±1.8	27.2±3.0	0.837
**Duration of analgesia (min)**	182.2±96.6	171.7±85.8	0.033
**Oxytocin**	88 (44%)	69 (40.6%)	0.508
**Mode of delivery, n (%)**			0.925
Spontaneous	191 (95.5%)	162 (95.3%)	
Instrumental	9 (4.5%)	8 (4.7%)	
**Conversion to caesarean section, n (%)**	47/247 (19.0%)	21/191 (11.0%)	0.021
**Indications for cesarean delivery, n (%)**			
Fetal distress	12/24 (25.5%)	8/21 (38.1%)	0.294
Prolonged labor	14/24 (29.8%)	7/21 (33.3%)	0.770
Cephalopelvic disproportion	13/24 (23.5%)	3/21 (14.3%)	0.230
Severe preeclampsia	3/24 (6.4%)	1/21 (4.8%)	0.793
Prenatal fever	5/24 (10.6%)	2/21 (9.5%)	0.889

Data are expressed as mean ± standard deviation or n (%). BMI = body mass index.

Table 1 demonstrated that parturients in the remifentanil group were taller in height and had a shorter duration of analgesia compared to those in the epidural group. Besides that, there was no statistically significant difference between the two groups as regards other demographic characteristics, mode of delivery and oxytocin utilization.

**Figure 2:**
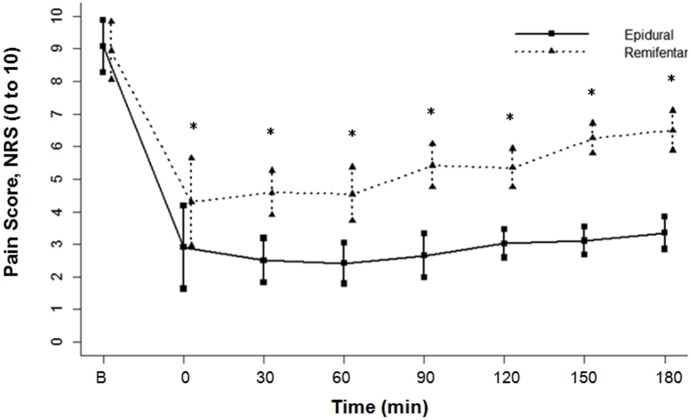
Comparisons in NRS pain scores between the two groups (Epidural group and Remifentanil group) at each time point. B represents baseline. NRS = numerical rating scale. *P<0.0001.

Comparing the two groups, pain scores were similar at baseline. After analgesic therapy, a significant decline in pain scores from baseline was discovered in both groups, and epidural pain scores decreased more at every given point in time (P<0.0001). One-hour treatment later, pain scores in the remifentanil group went steadily up but still inferior to the baseline. By comparison, the ascending tendency was minimal in the epidural group (Fig. 2).

**Figure 3:**
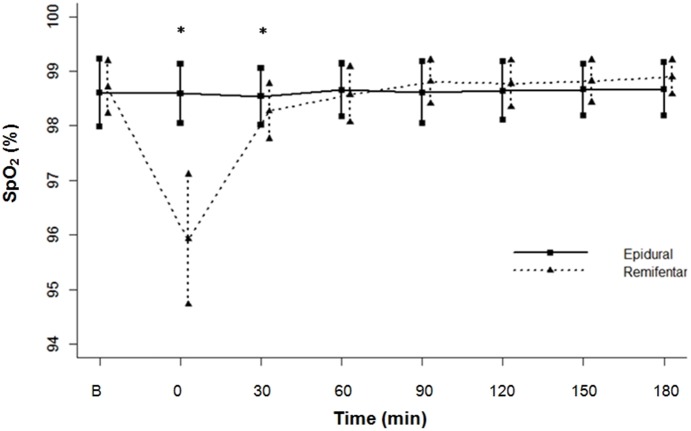
Comparisons in pulse oxygen saturation (SpO_2_) between the two groups.

Immediately after remifentanil analgesia, oxygen saturation reduced obviously compared to baseline (P<0.0001). And at that time point and 30 min after analgesia, mean SpO_2_ of the remifentanil group was lower than that of the epidural group, with significant difference (P<0.0001). Nevertheless, those who suffered from desaturation could recover their original state rapidly by deep breaths and supplementary oxygen. By contrast, oxygen saturation of those receiving epidural analgesia remained stable throughout the whole childbirth (Fig. 3). No respiratory depression (RR<9 breaths/min or SpO_2_<90%) was discovered in both groups.

**Figure 4:**
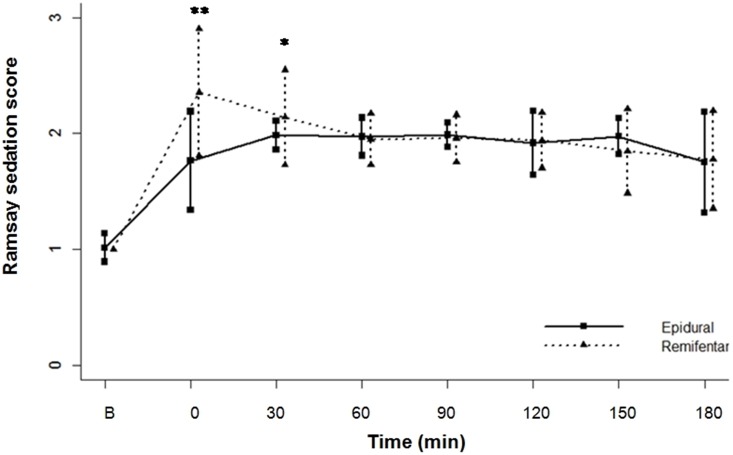
Comparisons in the Ramsay sedation score between the two groups (Epidural group and Remifentanil group) at each time point. B represents baseline. **P<0.0001, *P<0.001.

The Ramsay sedation scores were significantly higher in the remifentanil group immediately and 30 min after treatment (P<0.0001 and P<0.001, respectively) (Fig. 4). Six women following remifentanil regimen reached the maximum sedation score of 4.

The epidural group had a higher overall satisfaction score (3.8±0.4 vs. 3.7±0.6, P = 0.007) and pain relief score (2.9±0.3 vs. 2.8±0.4, P<0.0001) compared with the remifentanil group. Although more parturients receiving remifentanil reported nausea, vomiting and pruritus, the incidences of the above adverse reactions were similar between the two regimens. 21.8% of patients in the remifentanil group encountered dizziness, which was far higher than that in the epidural group (Table 2). Furthermore, logistic regression analysis demonstrated that nausea, vomiting were associated with oxytocin usage and instrumental delivery, and dizziness was relative to the type and duration of analgesia (Table 3).

**Table 2:** Quality of analgesia and Side effects.

	Epidural Group (n = 200)	Remifentanil Group (n = 170)	P value
**Overall satisfaction score**	3.8±0.4	3.7±0.6	0.007
4- very satisfied	167 (83.5%)	131 (77.1%)	
3- satisfied	33 (16.5%)	26 (15.3%)	
2- neutral (neither satisfied nor dissatisfied)	0	13 (7.6%)	
1- dissatisfied	0	0	
0- very dissatisfied	0	0	
**Pain relief score**	2.9±0.3	2.8±0.4	<0.0001
4- very good	0	0	
3- good	185 (92.5%)	132 (77.6%)	
2- moderate	15 (7.5%)	38 (22.4%)	
1- poor	0	0	
0- very poor	0	0	
**Dizziness**	2 (1%)	37 (21.8%)	<0.0001
**Nausea**	11 (5.5%)	13 (7.6%)	0.403
**Vomiting**	9 (4.5%)	11 (6.5%)	0.404
**Pruritus**	3 (1.5%)	4 (2.4%)	0.548

Data are expressed as mean ± standard deviation or n (%).

**Table 3:** Multiple logistic regression analysis with side effects (nausea, vomiting and dizziness) as the dependent variable.

	OR	95% CI	P value
**Nausea**			
Oxytocin	0.23	0.09–0.59	0.0025
Instrumental	0.21	0.06–0.74	0.015
**Vomiting**			
Oxytocin	0.23	0.08–0.66	0.0064
Instrumental	0.17	0.05–0.59	0.0056
**Dizziness**			
Type of analgesia	32.35	7.52–139.18	<0.00015
Duration of analgesia	1.01	1.001–1.009	0.02

OR = odds ratio; CI = confidence interval.

Neonatal data were summarized in Table 4. The analysis showed that there was no difference between the groups in relation to mean birth weight, Apgar scores and the incidence of abnormal FHR. FHR abnormalities included tachycardia, bradycardia, variable decelerations and late decelerations, and all were transient changes. The two groups were also similar with respect to the types of abnormal FHR. But umbilical pH and base excess of neonatus in the remifentanil group were significantly lower. Three newborns had 1 min Apgar scores <7, and all of them were from the epidural group. Two neonates were born after shoulder dystocia with Apgar score 4 and 5 for the 1^st^ minute, and 8 and 7 for the 5^th^ minute, respectively. The other had 1–5 min Apgar scores of 6–9 due to acute fetal distress. In addition, two neonates had an umbilical arterial pH<7.10, they were those who experienced shoulder dystocia in the epidural group. But no umbilical venous pH<7.10 was registered.

**Table 4:** Neonatal outcomes.

	Epidural Group	Remifentanil Group	P value
	(n = 200)	(n = 170)	
**Birth weight (g)**	3399.8±382.9	3439.8±371.8	0.444
**Apgar score**			
1 min	9.7±0.8	9.7±0.6	0.984
5 min	9.9±0.3	9.9±0.3	0.712
**Umbilical vein pH**	7.31±0.07	7.29±0.07	0.015
**Umbilical artery pH**	7.28±0.07	7.26±0.06	0.001
**Umbilical vein base excess (mol·l^−1^)**	−4.50±2.13	−5.08±2.21	0.011
**Umbilical artery base excess (mol·l^−1^)**	−4.96±2.66	−6.13±2.33	<0.0001
**Abnormal FHR changes, n (%)**	27 (13.5%)	33 (19.4%)	0.124
During the analgesia period, n (%)	16/27 (59.3%)	24/33 (72.7%)	0.271
- Tachycardia, n (%)	3/16 (18.8%)	5/24 (20.8%)	0.601
- Bradycardia, n (%)	6/16 (37.5%)	11/24 (45.8%)	0.872
- Variable decelerations, n (%)	5/16 (31.3%)	7/24 (29.2%)	0.888
- Late decelerations, n (%)	2/16 (12.5%)	1/24 (4.2%)	0.327

Data are expressed as mean ± standard deviation or n (%). FHR = fetal heart rate.

## Discussion

This research work shows that epidural analgesia appears to afford more preferable analgesia effect than remifentanil IVPCA. Pain scores reported from the epidural group were significantly lower at each set time-point (0, 30 min, 60 min, 90 min, 120 min, 150 min and 180 min after treatment), and epidural regimen produced more persistent contribution on labor analgesia. In relative terms, administration of remifentanil just had moderate pain reduction with gradual elevation of pain scores as the labor progressed. These findings were consistent with other recent studies [6], [8], [13]–[18]. Our previous meta-analysis has also demonstrated that there were higher pain scores at 1 h and 2 h for patients with remifentanil IVPCA compared with those receiving epidural analgesia [10]. At the beginning of remifentanil IVPCA, pain relief was still satisfactory because of its rapid onset. Progressive pain of uterus systole with the progress of labor and/or a tolerance to remifentanil after continuous use were probably responsible for the later rising pain scores in the remifentanil group [7], [19]. Obviously, local anaesthetic by epidural had more control over the pain stress.

Although 92.4% of parturients with remifentanil IVPCA expressed satisfaction with analgesic effect (very satisfied: 77.1%, satisfied: 15.3%), the overall satisfaction scores and pain relief scores were lower in the remifentanil group, which seemed distinguished from those seen in other researches. Douma et al. [7] found there was no obvious distinction in satisfaction scores after delivery between the remifentanil group and the epidural group. Similarly, in the study of Stourac et al. [20], the level of the parturients’ satisfaction with analgesia was similar both in the EA group and in the rPCA group (P = 0.24). One possible explanation for this was that these assessments carried subjective criteria to result in the difference. In addition, interindividual variation in the response to opioid [21], [22] and the different administration schedules we adopted were likely to account for the disparity.

Up to the present, the most suitable dosage regimen of remifentanil for labour still retains a controversial subject [23]. While the regimen without background infusion has been reported to produce superior effect on obstetric analgesia [14], [24]–[28], recent researches indicated that fixed small PCA boluses with alterable infusion rate could provide effective analgesia with fewer adverse reactions [23]. Our dosage regimen of remifentanil (0.04∼0.05 µg·kg^−1^·min^−1^ infusion and 0.4 µg·kg^−1^ PCA bolus) made reference to the previous studies and experiences from our institution. In clinical practice, we found remifentanil presented a delayed peak effect in spite of its quick onset. Thus PCA bolus alone could not act urgently to keep uterine contraction pain under control. Furthermore, a dose-related risk of respiratory depression and excessive sedation exist after remifentanil bolus injection, which has been discovered in healthy volunteers [29]. Some studies have recommended the bolus dose of 0.4 µg·kg^−1^ can be used effectively and securely [6], [8], [18], [26], [30], the infusion rate more than 0.05 µg·kg^−1^·min^−1^ may be connected with higher incidence of side effects [14].

In this study, a marked drop in maternal SpO_2_ appeared within 30 min after using remifentanil. It may be related to a transient respiration inhibition at the onset of remifentanil. The oxygen desaturation (defined as SpO_2_<95%) episode persisted only for a brief duration, which could be reversed by deep breathing and oxygen inhalation through nasal tube. The incidence of oxygen desaturation we observed was 9.4%, far below other reported rates (40%∼74%) [14], [16], [18], [31]–[33]. Perhaps that had something to do with preventive oxygen supply. And, continuous SpO_2_ monitoring and bedside-monitor of the anaesthetist or midwife also contributed a great share in preventing desaturation, since hypoxia caused by remifentanil might still occur even in the situation of oxygen supply [8]. Besides, dehydration or exhaustion along with the application of remifentanil also could aggravate respiratory depression [34], so adequate transfusion treatment in advance is recommended.

Despite a higher level of sedation in the remifentanil group, all of patients could be awakened easily by a loud voice or the next uterine contraction pain. 21.8% of patients receiving remifentanil reported dizziness, and we noted that a longer duration of remifentanil analgesia was more likely to cause dizziness. We speculated it might be related to a certain degree of cumulative effect. Yet, nobody complained uncomfortable for it. That being said, one-to-one nursing still needs to be ensured.

The frequencies of nausea, vomiting and pruritus were similar between the two groups, which were consistent with the results from previous studies [6], [8], [32], [35]. Our analyses pointed out that some obstetric factors such as the usage of oxytocin and forceps may be associated with nausea and vomiting which are common during delivery. Studies showed that the occurance of nausea and vomiting is correlated to the degree of hypotension [36]. Higher doses of oxytocin or forceps delivery may lead to sudden haemodynamic change.

The impact of remifentanil on newborns has become a common concern. Neonatal outcomes observed in our study stayed at an acceptable level. Removed from two neonates with shoulder dystocia and one with acute fetal distress, other Apgar scores were normal. Besides, there were no significant differences in the incidence or types of abnormal FHR between the two groups. From the current data, systemic remifentanil seemed have no serious effect on FHR. However the fact that opioids have the capability of producing FHR abnormalities [6] cannot be taken lightly. We guessed close monitoring and routine oxygen supplementation should play a part in this. The mean umbilical cord gases in both groups were kept in normal range as well, whereas umbilical arterial/venous pH and base excess were lower in the remifentanil group. Our findings seemed to be different from previous studies [6], [7], [9], [14], [26], [35], which found no effect from remifentanil on neonatal outcomes including umbilical pH and base excess. Despite the fact that remifentanil is easy to be metabolized in the neonates [30], [37], it still has some influence on neonatal status. Therefore, we recommended neonatal resuscitation should be in train before birth for the neonates whose mothers have received remifentanil. 2010 American Heart Association Guidelines for Neonatal Resuscitation suggested that at least 1 person who must be capable of initiating resuscitation, including administration of positive pressure ventilation and chest compressions, should present at every delivery [38]. 2011 Chinese Neonatal Resuscitation Guidelines also have the same recommendations [39]. In our hospital, every delivery room keeps the necessary equipments and medications for resuscitation available. All practitioners in the delivery rooms including midwives are required to know the whole resuscitation process and have skills to perform the initial steps of resuscitation. The initial steps (about 60 s), which are called “the Golden Minute”, will win precious rescue time for skilled personnel’s arrival. Coordination between obstetrics and pediatrics, regular training, adequate preparation and prompt initiation of support are a forceful guarantee for successful neonatal resuscitation.

The demographics of two groups were matched except for height and duration of analgesia. The conversive rate and the indications of caesarean section are similar between the two groups. However, we have not been able to determine whether intravenous remifentanil for labour analgesia is helpful with lowering the conversive rate due to limited samples of our present study. As for the conversive rate of caesarean section in our study (15.5% [68/438]), it was really lower than figures reported in other studies. Ismail MT et al. [40] reported that the conversive rate in their study was 24.3%. We guessed it might be related to our inclusion criteria that only healthy primiparas without any obstetrics complications can be included. These patients were less likely to undergo caesarean section. Moreover, one-to-one bedside care, continuous monitoring, timely management and treatment may be also crucial to reduce the conversive rate of caesarean.

As a result of our retrospective study, the probability of missing data and selection bias is unavoidable. It is also the main limitation of our study. For instance, some data relating to blood pressure, heart rate, breathing rate and the quantities of drugs were not recorded completely and missing. A further prospective study will designed to refine the data. However, we believe the omissions and bias may be not big because we adopted a standardized analgesic procedure for labour and a detailed electronic recording system.

In this retrospective study, we confirmed that remifentanil IVPCA produced an observable improvement in pain scores, though not quite as efficacious as epidural analgesia. Furthermore, we also have reached some different conclusions from previous researches. For example, patients’ satisfaction scores and neonatal umbilical cord gases in the remifentanil group were lower than those in the epidural group. As a systemic opioid, sedation, dizziness and desaturation were inevitable during using remifentanil. Fortunately, these adverse reactions were temporary and the effects on neonatal outcomes were small. We suggest that remifentanil analgesia can be implemented as an option for pain relief during childbirth under the precondition of ensuring one-to-one bedside care, continuous oxygen saturation monitoring, oxygen supply up front, and immediate availability of neonatal resuscitation.
